# Rhythmicity, Recurrence, and Recovery of Flagellar Beating

**DOI:** 10.1103/PhysRevLett.113.238103

**Published:** 2014-12-02

**Authors:** Kirsty Y. Wan, Raymond E. Goldstein

**Affiliations:** Department of Applied Mathematics and Theoretical Physics, Centre for Mathematical Sciences, University of Cambridge, Wilberforce Road, Cambridge CB3 0WA, United Kingdom

## Abstract

The eukaryotic flagellum beats with apparently unfailing periodicity, yet responds rapidly to stimuli. Like the human heartbeat, flagellar oscillations are now known to be noisy. Using the alga *C. reinhardtii*, we explore three aspects of nonuniform flagellar beating. We report the existence of rhythmicity, waveform noise peaking at transitions between power and recovery strokes, and fluctuations of interbeat intervals that are correlated and even recurrent, with memory extending to hundreds of beats. These features are altered qualitatively by physiological perturbations. Further, we quantify the recovery of periodic breaststroke beating from transient hydrodynamic forcing. These results will help constrain microscopic theories on the origins and regulation of flagellar beating.

Patterns of coordinated movement in living organisms, such as walking, running, and galloping, may be variable yet simultaneously stable. Such repetitive dynamics are distinguished by their reproducibility, longtime sustainability, and robustness to moderate perturbations. In the precise, rhythmic beating of the flagella of the alga *Chlamydomonas* we find remarkable living oscillators that fulfill these three criteria. The synchronous beating of its twin ~10 *μ*m long flagella allows *Chlamydomonas* to swim a fast breaststroke [1]. At ~60 Hz, its flagellar oscillations are self-sustained—repeated mechanochemical cycles continuously supply energy to motor dyneins within flagellar axonemes [2]. The stepping action of individual motors is intrinsically stochastic [3], and yet, beating can nevertheless persist, resilient against a cacophony of biochemical and background fluctuations. In assessing the fidelity or robustness of a biological oscillator, the stability and rhythmicity of its oscillations serve as prime indicators: one might identify pathological gaits of human walking from measures of cycle stability [4], determine the phase-dependent response of circadian clocks using external stimuli [5], or infer the health of a human heart from the variability of interbeat intervals [6,7]. While periodic oscillations of beating flagella are correlated with a cell’s responses and sensitivity to its environment, study of these features remains inchoate [8–13].

Here, drawing on data from a large population of cells (~100), we examine fluctuations in beating due to perturbations that are (a) continuous, or (b) transient. Case (a) encompasses contributions from sources over which the experimenter has little control: background thermal noise, intracellular biochemical processes [14], or even photon irradiance [15]. We find that flagellar dynamics are stable to these weak fluctuations, but waveform noise displays an intriguing phase dependence, or *rhythmicity*. Beat-to-beat intervals form time series exhibiting fractal structure, and successive beats may remain correlated for many seconds, even displaying oscillatory correlation (*recurrence*). Yet in cells placed under physiological stress, measured time series signal much more erratic and variable flagellar beating dynamics. For (b), we inject fluid impulses near a beating flagellum and examine the postperturbation relaxation to the stable limit cycle of the breaststroke. This *recovery* from beating disruption is a crucial property of viable cilia and flagella.

To permit longtime, in-focus visualization of flagellar dynamics, wild-type cells (strains CC124 and CC125, *Chlamydomonas* Center) were individually caught and fixed by micropipette micromanipulation (Patchstar, Scientifica, UK) with gentle suction [8,11]. High-speed images (Fastcam SA3, Photron USA and Phantom v311, Vision Research) of beating flagella were captured at 2000–3000 frames*=*s—at least 1 order of magnitude above the natural beat frequency. Continuous recordings (1–10 minutes) were taken for each cell, from which ~1–5 × 10^3^ contiguous beat cycles could be extracted. Recordings were conducted under conditions that appropriately mimic a cell’s natural daytime habitat, namely, white light illumination (halogen lamp), and hence some phototactic response is expected [16]. Pixel coordinates that track the flagellum in each frame were converted to spline fits and used to generate time series.

Automated waveform tracking gives unprecedented spatiotemporal resolution [11], which over thousands of cycles allows a determination of the spatial reproducibility of the beating. Relative to a reference axis, angles *θ*(*t*) traced by a point at fixed arclength [17] [Fig. 1(a)] are projections of the multidimensional dynamics. The point cloud $\left(\theta ,\dot{\theta }\right)$ maps the attracting region around a limit cycle Γ—approximated numerically. Progression through each cycle was charted by associating the 2D flagellum center-line **f**(*t*_*i*_) at time *t*_*i*_ with a uniformly rotating phase *ϕ* = *ω*_0_*t* defined from the polar angle $\varphi =\tan^{-1}(\theta -\langle \theta \rangle )/(\dot{\theta }-\langle \dot{\theta }\rangle )$ using the transformation *ϕ* = *ω*_0_ ∫ (*dφ/dt*)^−1^*dφ*. Ratio distributions are approximated using Fourier series [11]. Trajectory crossings *C* = {**x**_*n*_∶*ϕ*(*𝒫*^*n*^(x_*n*_)) = *ϕ*_0_; *n* = 1, 2, 3, …} at fixed *ϕ* = *ϕ*_0_ correspond to iterations of a Poincaré return map *𝒫*. We computed, for each cell and 50 subdivisions of [0, 2*π*], eigenvalues of the Jacobian matrix of derivatives $J={D𝒫|}_{x^{*}}$ taking x* = ⟨x⟩_x∈*C*_ and fitting to the bilinear model (**X**_*n*+1_ − X*) = *𝒯* (**X**_*n*_ − X*). The distribution of computed eigenvalues [Fig. 1(b)] is particularly dense on the real line. All eigenvalues have magnitude less than unity, fulfilling our intuition that limit cycles corresponding to the breaststroke gait are stable.

To examine the phase dependence in the noise suggested by Fig. 1(c), we appeal to the full dimensionality of the waveforms. The set $S_{k}=\left\{f\left(t_{j}^{k}\right)\right\}$, where $\left\{j:{\phi (t)|}_{t=t_{j}^{k}}=\phi_{k}\right\}$ for phases *ϕ*_*k*_ = 2*πk*/50, *k* = 1; …, 50, groups periodic waveforms at an equivalent phase [Fig. 2(a)]. We measure the dissimilarity between **f** ∈ *S*_*k*_ and an average waveform $f_{k}^{*}$ by a (discrete) *Fréchet distance*

(1) $$\delta_{F}\left(f,f_{k}^{*}\right):=\underset{U}{\min}\underset{(p,q)\in U}{\max}∥p-q∥,$$

with tracked waveforms approximated by polygonal curves corresponding to ordered vertices *σ*(**f**) = (**p**_1_; …; **p**_*m*_) and $\pi \left(f_{k}^{*}\right)=\left(q_{1},\ldots ,q_{n}\right)$ (*m,n* ∈ ℤ). and

$$U=\left\{\left(p_{u_{i}},q_{v_{j}}\right)\in \sigma (f)\times \pi \left(f_{k}^{*}\right)\;(i,j=1,\ldots ,J)\right\},$$

comprising *J* pairs of vertices which are complete [for every **p** ∈ *σ* there exists *i, j* with (**p**_*i*_, **q**_*j*_) ∈ *𝒰* and **p** = **p**_*i*_; a similar statement holds for **q** ∈ *π*] and ordered (*u*_*i*+1_ = *u*_*i*_ or *u*_*i*+1_ = 1 + *u*_*i*_, and *v*_*j*+1_ = *v*_*j*_ or *v*_*j*+1_ = 1 + *v*_*j*_). The computation is performed recursively, in 𝒪(*mn*) time [18]. At each phase *δ*_*F*_ gauges waveform noise in the periodic formation of the flagellum shape [Fig. 2(b)] and is minimized during recovery strokes (≲1.7%—a value comparable to measurement noise), and it is maximized at the transitions between power and recovery strokes (≳10.8%).

In a classic eukaryotic flagellum, beating emerges from periodic, selective activation of motor dyneins that cross-link internal filaments [2]. At putative switch-points between power and recovery strokes [19], geometrically opposed groups of dyneins detach on one side and reattach at the other to their respective microtubule tracks until beating direction is reversed. Thus, high waveform noise correlates with a large number of activated dyneins.

The timing of flagellar strokes is determined by the microscale action of dyneins, which in turn governs the frequency and amplitude of the beat. Here we partition flagellar positions by phase, averaging two different Poincaré sections to obtain the instantaneous period *T*_*n*_ and the frequency *ν*_*n*_ = 1*/T*_*n*_, indexed by beat number *n*. From the data, we approximated the *n*th-cycle beat envelope by its *alpha shape* [20], which generalizes the concept of a convex hull [Fig. 2(c)]. Accuracy in the computed *alpha shape* area *α*_*n*_ is defined up to discs of radii 5 pixels ≈ 1.11 *μ*m. We find *T*_*n*_ and *α*_*n*_ to be strongly correlated. Denoting by ⟨·⟩ an average over beat cycles and plotting Π = *T*_*n*_/⟨*T*_*n*_⟩ vs *A* = *α*_*n*_/⟨*α*_*n*_⟩ reveals directional but very noisy scatter. A similar correlation has been found independently [13]. To characterize this directionality, we compute the matrix

(2) $$\text{Cov}[\text{Π},A]=\left(\begin{array}{c} \langle TT\rangle \;\langle TA\rangle \\ \langle AT\rangle \;\langle AA\rangle \end{array}\right),$$

where *𝒯* = Π − ⟨Π⟩ and *𝒜* = *A* − ⟨*A*⟩. From the time series for each cell *i* we estimate *𝒯/𝒜* by *γ* = tan^−1^(*v*_2_*/v*_1_), where (*v*_1_, *v*_2_) is the principal eigen-vector direction [Fig. 2(d)]. We find $\bar{\gamma }\sim (0.264\pm 0.146)$ ~ (0.264 ± 0.146) rad where the bar denotes an ensemble average over multiple cells and, correspondingly, a dimensional ratio of increments $\bar{r}\approx 39.7\pm 31.0\;\mu {\text{m}}^{2}/\text{ms}$, where *r* = ⟨*α*_*n*_⟩ / ⟨*T*_*n*_⟩ × 1/ tan(*γ*). Assuming a flagellum “wing-span” of 10 *μ*m during the power stroke, this is equivalent to a velocity scale of *δℓ/δT* ~ 4 *μ*m*/*ms for an effective amplitude *ℓ*. A rodlike flagellum of length *ℓ* produces a motive force *F* ~ *ηT*^−1^*ℓ*^2^ and a power density *P/ℓ*, where *P* ~ *ηT*^−2^*ℓ*^3^ (where *η* is the medium viscosity); that amplitude and frequency are inversely correlated suggests a constancy of force, power production, or both by axonemal motors and is often assumed without proof in certain bead-on-spring models of beating cilia. Fundamentally, hydrodynamic synchronization in coupled ciliary arrays also necessitates that (within a physiologically relevant regime) decrease in beat frequency accompanies increase in amplitude [21], as we have demonstrated here.

The association of oscillatory dynamics with a well-defined frequency does not *a priori* imply stability. Stable flagellar beating, as we have now established for the canonical *Chlamydomonas* breaststroke, does not generalize to all flagellate species nor to *Chlamydomonas* cells that are physiologically “abnormal.” For instance, the time series *ν*(*t*) in Fig. 3(a) are representative of flagellar beat frequency fluctuations in a number of scenarios of interest. In case 1 we initiated complex calcium fluctuations and repair processes in a cell [22] by mechanical deflagellation of one flagellum; *ν*(*t*) was then measured for the remaining flagellum, which continues to beat as the amputated flagellum is regrown within 1–2 h. A heat shock treatment was used in case 2 to disrupt enzymatic pathways [23] in which cell cultures were immersed in a 35° water bath for ten minutes prior to experimentation. Case 3 is a control cell. Cells in case 4 were subject to a frontally directed flow, controlled by a syringe pump (PHD2000, Harvard Apparatus). Filtering the illumination light (> 620 nm filter) leads to persistent light-adaptation processes and frequency drift [10]; this is case 5. Noisy flagellar dynamics are thus a directly quantifiable measure of a cell’s physiological state.

Even control cells [Fig. 3(a), case 3] are subject to persistent, weak environmental fluctuations that feedback modulate flagellar beating. Measured beat frequencies in the two flagella of a given cell agree with remarkable precision [Fig. 3(b), inset]. From time series *b*(*t*) of the interbeat intervals, we construct the statistic *C*(*τ*) = ⟨ *b*(*t* + *τ*)*b*(*t*) − ⟨*b*⟩^2^⟩, where ⟨·⟩ denotes a time average. The decay of *C*(*τ*) was found to be unexpectedly slow, and in many cases even oscillatory [Fig. 3(c)]—suggestive of an underlying periodic process with noise. Let *b*(*t*) = *b*_0_(1 + *β*(*t*)) cos (*ω*_0_*t* + *ϕ*(*t*)), where *b*_0_ and *ω*_0_ are the averaged amplitude and frequency of oscillations and *β*(*t*), *ϕ*(*t*) are independent functions respectively characterizing phase and amplitude noise. We assume that *β*(*t*) is stationary and that *ϕ*(*t*) is a Brownian motion with ⟨*ϕ*(*t*)⟩ = 0 and ⟨*ϕ*(*t*)^2^⟩ = *Dt*. The autocorrelation is

(3) $$\tilde{C}(\tau )=\frac{b_{0}^{2}}{2}\left[1+C_{b}(\tau )\right]{\text{e}}^{-D|\tau |}\cos\left(\omega_{0}\tau \right),$$

where *C*_*b*_ is the covariance of *β*(*t*). For a sample cell we fit *C*(*τ*) using Eq. (3) with an empirical function *C*_*b*_(*τ*) = *βe*^−|*τ*|/*ξ*^ [Fig. 3(c), inset], yielding *b*_0_ = 0.157, *D* = 0.002, *ω*_0_ = 0.016, *β* = 9.928, and *ξ* = 1.85. In particular we find a time scale for the periodicity of slow oscillations: 2*π/ω*_0_ = 392 beats, or 6.01 s. Sampled over 65 cells, the average form of *C*(*τ*) takes ~250 beats for the correlation to reverse sign and persists over ~1000 beats, or ~15 s.

Our *b*(*t*) time series possess fractal structure and are correlated across multiple scales. In the first instance we can derive a scalar measure *a* via a detrended fluctuation analysis to characterize an individual time series [24], as follows. Construct first the integrated signal $B\left(t_{j}\right)=\sum_{i=1}^{j}\left(b\left(t_{i}\right)-\langle b\rangle \right)$, (1 ≤ *j* ≤ *L*). Then for *K* sections {*I*_*i*_ ≔ [*t*_*i*_, *t*_*i*+1_], *t*_*i*_ = *iL/K, i* = 1, 2, …, *K* – 1}, each of size *N* = *L/K*, the local trend in *B* is computed at the *i*th section [let *B*_*N*_(*t*_*i*_) be the least-squares linear fit to data points *B*(*t*_*i*_ ∈ *I*_*i*_)]. The fluctuation

(4) $$F(N)=\sqrt{\frac{1}{L}\sum_{i=1}^{L}{(B\left(t_{i}\right)-B_{N}\left(t_{i}\right))}^{2}}$$

is computed at multiple scales and a power-law scaling *F*(*N)* ~ *N*^*a*^ is obtained. We calculated *a* = 0.83 ± 0.10 [for 67 cells, *𝒪*(10^3^) successive beats each]. This persistent positive correlation is lost upon randomly permuting *b*(*t*) (each time averaging over 10 shuffles), which yields *a* = 0.48 ± 0.03, consistent with white noise.

The frequency (and hence synchrony) of flagellar beating is controlled at a biomolecular level by calcium [25,26]. Previously we found that the flagella of free-swimming *Chlamydomonas* switch stochastically [8] between synchronous and asynchronous beating (drifts) on a time scale of ~10 s, and we suggested this may be due to calcium fluctuations which affect *cis* and *trans* flagella differentially [11]. Our present discovery of slow oscillations in flagellar beat frequency might then relate these transitions in beating modes to stochastic crossings of a putative calcium threshold. Fluctuations in the cytosolic calcium of *𝒪*(*s*) have been measured *in vivo*, in *Chlamydomonas* cells ballistically loaded with calcium dyes [22].

Sudden elevations in intracellular calcium can be triggered by activation of either photoreceptors in the eyespot [27] or mechanosensitive channels in the membrane [28], leading to altered flagellar beating. By perturbing a beating flagellum with manually induced pulses of fluid from a second pipette (which delivers ~100 pN forces, according to particle image velocimetry measurements), we can compute the attractor strength *σ* of flagellar oscillations [Fig. 4(a), panels 1–3]. Limit cycles and phases are defined from tracked waveforms as previously (Fig. 1). The preperturbation cycle **r**_*L*_ was chosen for reference purposes. If the perturbed trajectories **r**(*t*) evolving in time *t* contract linearly towards the stable attractor, then at a representative phase *ϕ* = *ϕ*_0_,

(5) $$\sigma =\frac{1}{\tau_{\infty }-\tau_{0}}\ln\frac{\left|r\left(\tau_{\infty };\phi_{0}\right)-r_{L}\left(\phi_{0}\right)\right|}{\left|r\left(\tau_{0};\phi_{0}\right)-r_{L}\left(\phi_{0}\right)\right|},$$

where *τ*_0_ is chosen at maximum deviation and *τ*_∞_ when **r** first returns (and remains) within an acceptance band about **r**_*L*_. Averaging multiple experiments, we find *σ* ~ 2.94 ± 1.72 s^−1^ or *σ*^−1^ ~ 20.4 beats. Thus normal flagellar beating can readily (and in characteristic time) recover from moderate hydrodynamic disturbances which mimic that which microalgae encounter in their native habitats. If local perturbation of one flagellum transiently elevates intra-cellular calcium, the observation of altered beating of both flagella in a coupled pair [Fig. 4(d)] is consistent with differential *cis*-*trans* flagellar calcium response, or dominance [11]. This rapid loss of biflagellar synchrony implicates internal biochemical control of normal breast-stroke coordination [Fig. 3(b), inset]; in contrast, the beating of flagella belonging to different cells can be synchronized solely by the hydrodynamics [29].

Through dynamic high-resolution tracking, the rhythmicity of eukaryotic flagellar oscillations was revealed and the nature of flagellum noise explored. We demonstrated significant spatiotemporal correlation in the beating dynamics and suggested that while variations on time scales of beat cycles may be due to intrinsic motor stochasticity, long-range correlations in beat frequency may be signatures of *in vivo* biochemical signalling via second messengers such as calcium [30]. Indeed, calcium governs ciliary beating in many different organisms [31–33]; oscillatory calcium dynamics would vastly improve specificity, allowing signals to integrate without sustained rise. It would be interesting to examine the noise spectrum of beating in artificial or reconstituted flagella, where feedback regulation would take on a very different form.

We thank M. Polin, K. C. Leptos, and P. Holmes for the discussions. Financial support is acknowledged from the Engineering and Physical Sciences Research Council, European Research Council Advanced Investigator Grant No. 247333, and a Senior Investigator Award from the Wellcome Trust.

## Figures and Tables

**Fig. 1:**
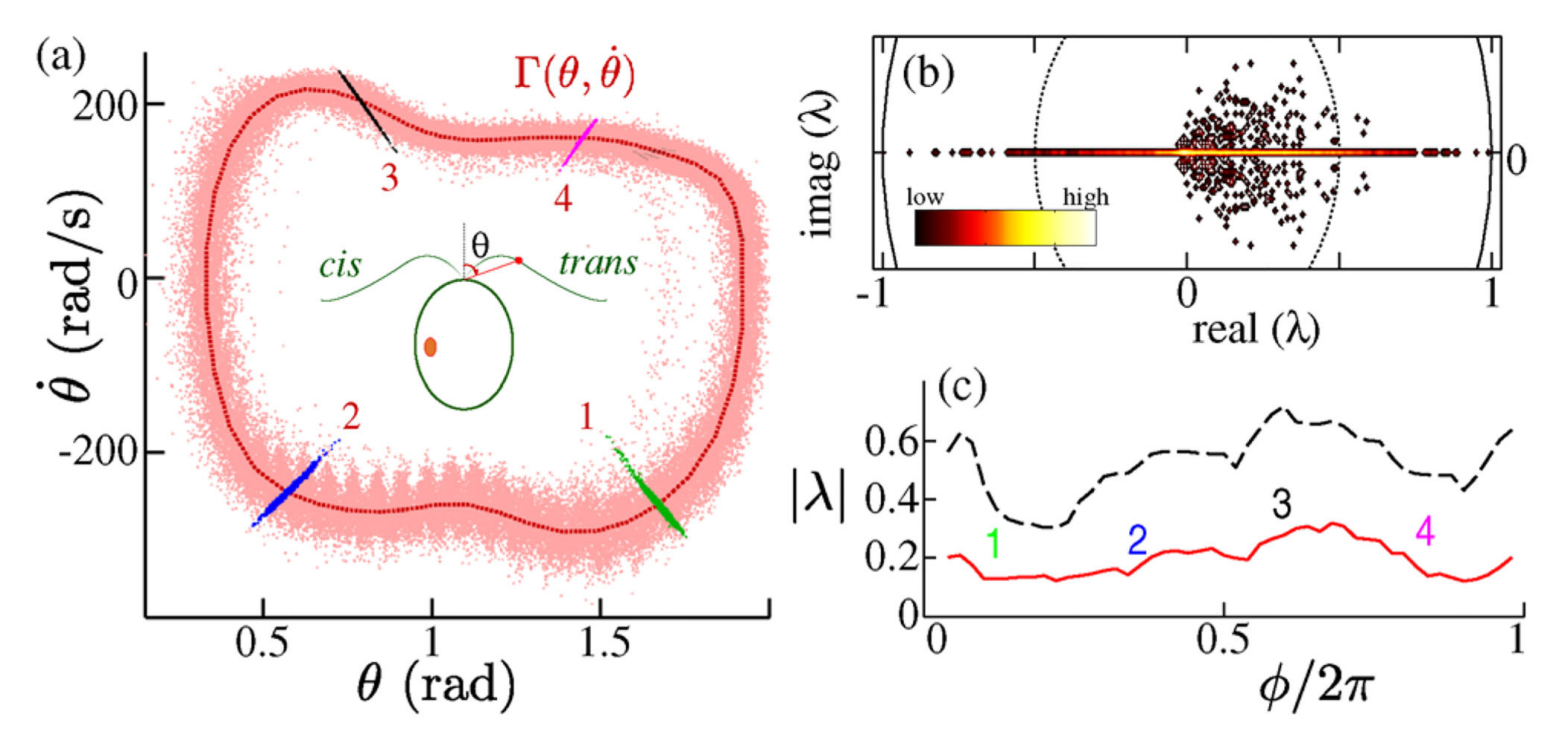
Noisy flagellar limit cycles. (a) Trajectories in $\left(\theta ,\dot{\theta }\right)$ space at fixed arclength [= (2*/*7) of the total flagellum length]. Four Poincaré sections are highlighted. For the population (*n* = 48), (b) shows an accumulated density map of Floquet multipliers {*λ*} computed at different phases, while in (c) the distribution of |*λ*| is characterized by its mean (solid line) and 95th percentile (dashed line).

**Fig. 2:**
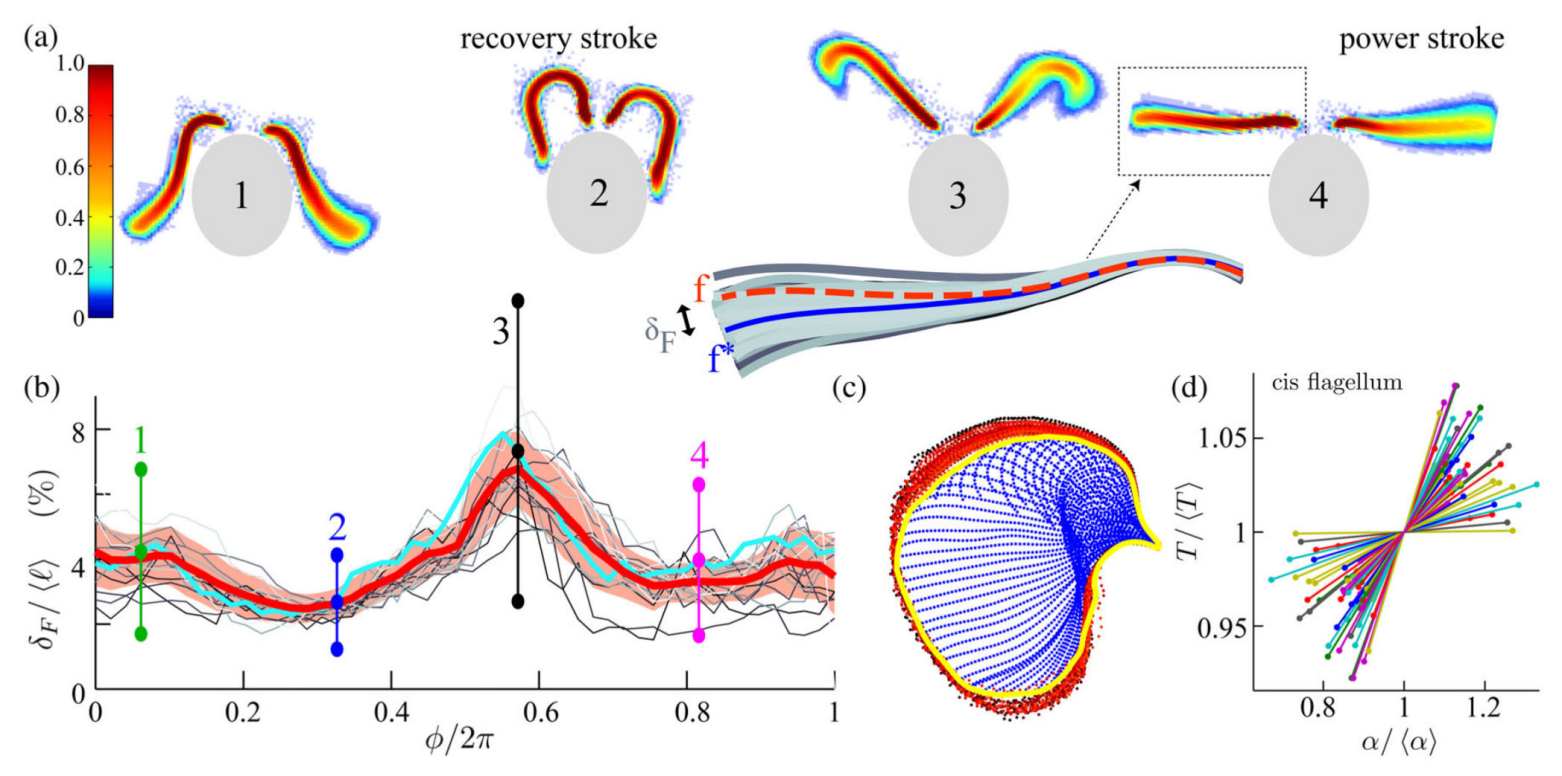
Noisy flagellar waveforms. (a) Overlaid waveforms at phases 1–4 [Fig. 1(a)], colored by recurrence. Isophase waveforms **f** coalesce in a band about an average shape f*. (b) Length-normalized Fréchet distance *δ*_*F*_(f, f*) / *ℓ* computed for multiple cells, showing phase-dependent noise. (Cyan) Average over *𝒪*(10^3^) beat cycles for a single cell; error bars: one s.d. from mean. (Red) A multicell average; shading: one s.d. from mean. (c) Discretized points (blue) along a flagellum define an area per beat via an alpha shape (yellow) which fluctuates over successive beat cycles (red). (d) Per-beat area *α* and per-beat period *T* are strongly correlated. Individual lines summarize the per-cell noisy scatter.

**Fig. 3:**
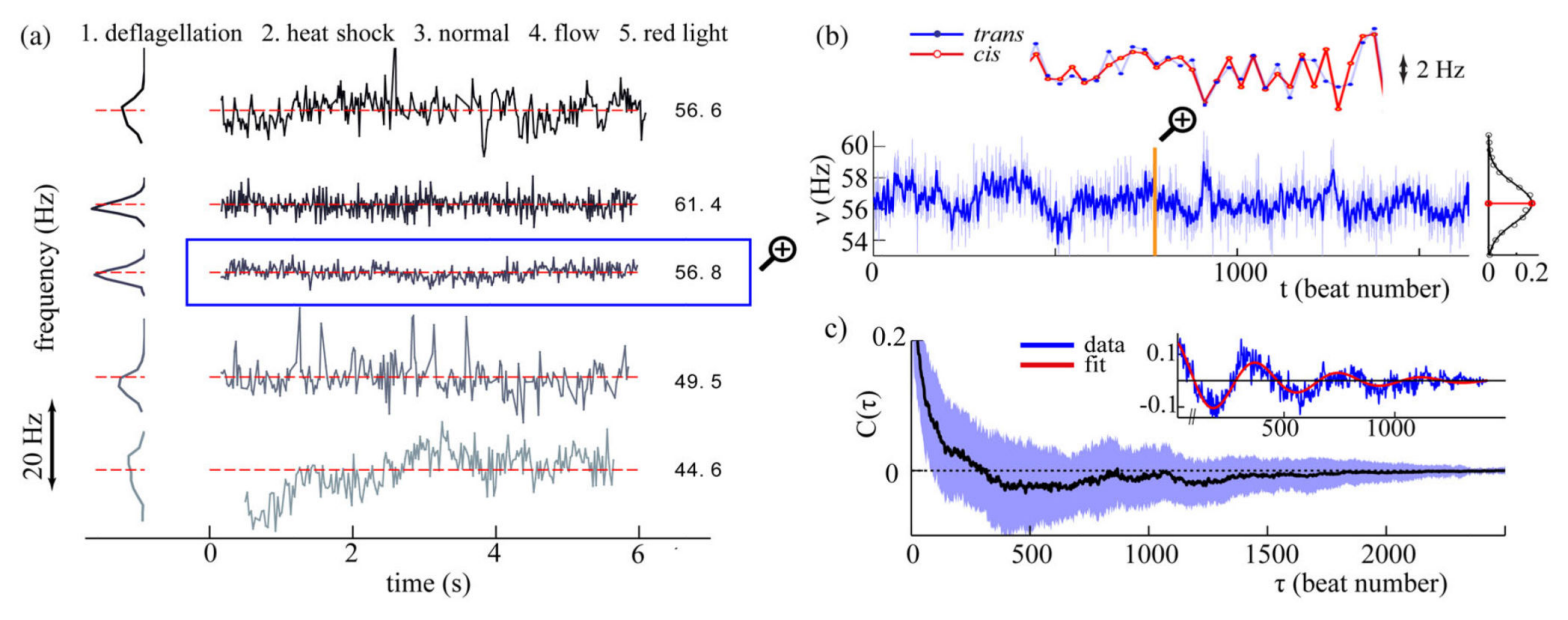
Correlations in flagellar beating. (a) Signatures of interbeat frequencies *ν*(*t*) in the flagella of cells, observed in a number of scenarios (1–5, see main text). (Time series have been displaced vertically, with mean frequencies as labeled.) (b) Long-range fluctuations in *ν*(*t*) are observed. For a control cell (case 3), the filtered signal is superimposed with the raw data, and its probability distribution function fit to a Gaussian function. *Cis* and *trans* flagella of the same pair exhibit perfect frequency locking, highlighting the accuracy of the measurement technique. (c) Decay of autocorrelation in interbeat intervals *b*(*t*) ≔ 1/*ν*(*t*), showing the population average (solid line) and one s.d. from the mean (shaded). (Inset) Parametric fit to a sample *C*(*τ*).

**Fig. 4:**
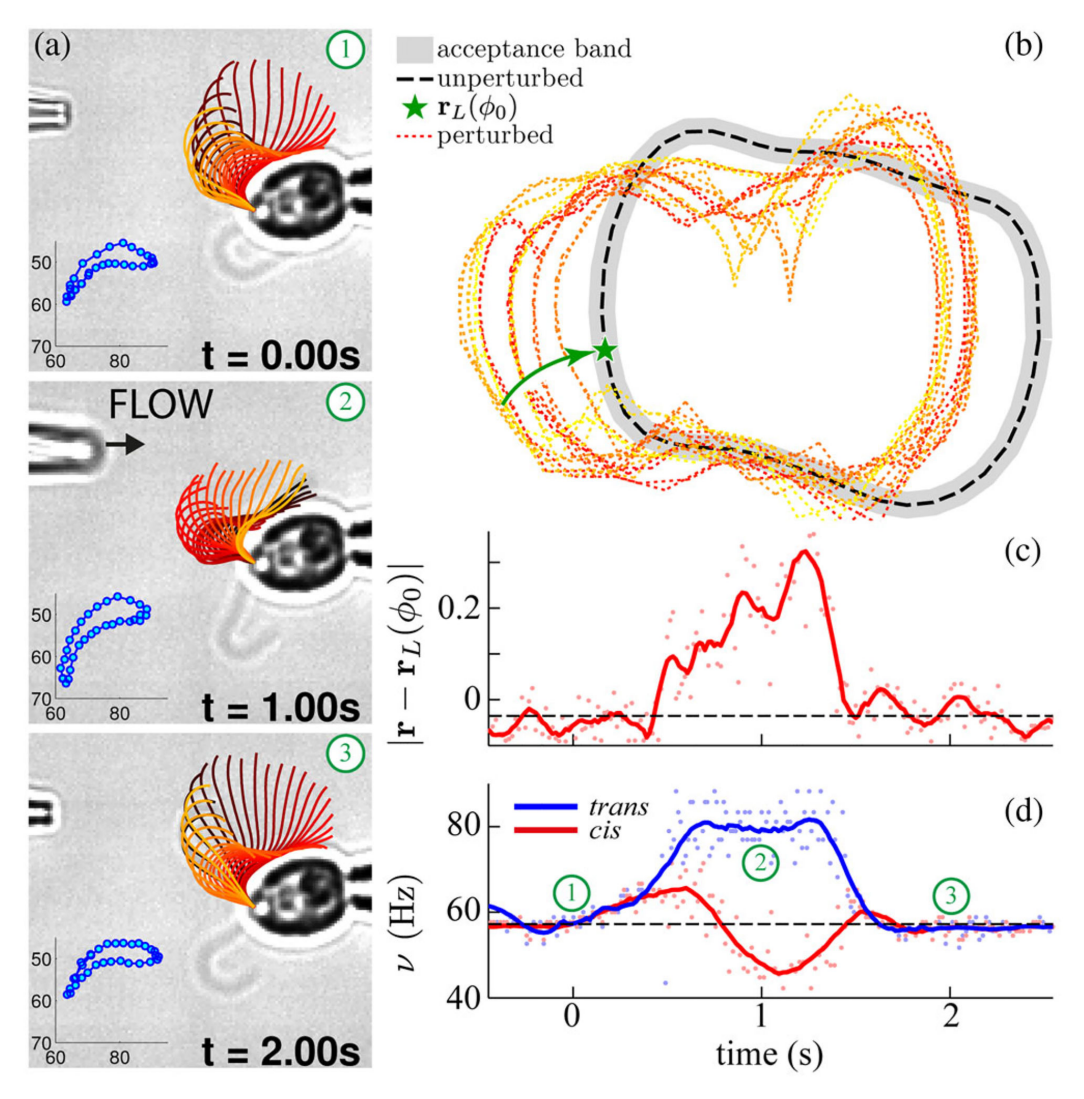
Stability to perturbations. (a) Fluid is injected from a second pipette (arrow). Waveform sequences for the *cis* flagellum only are shown (1–3). (Insets) *x*-*y* coordinates of a reference point at a fixed arclength. (b) Trajectories veer off the preperturbation limit cycle during one perturbation event. This deviation is sampled at fixed phase as a function of time (c), which accompanies marked changes in the beat frequencies of both flagella (d).
